# Exploring Chemical Basis of Toxicity Reduction for Processed Roots of* Stellera chamaejasme* L. Using Ultraperformance Liquid Chromatography–Triple Quadrupole Tandem Mass Spectrometry

**DOI:** 10.1155/2019/4854728

**Published:** 2019-03-06

**Authors:** Wei Yang, Xiaoli Ma, Ludi Wang, Mengmeng Wei, Shuyao Wang, Siyang Wu, Chen Kang, Yingfei Li

**Affiliations:** ^1^Center for DMPK Research of Herbal Medicines, Institute of Chinese Materia Medica, China Academy of Chinese Medical Sciences, Beijing 100700, China; ^2^College of Traditional Chinese Medicine, Hebei University, Hebei 071000, China

## Abstract

Some herbal medicines are treated with various processing methods to ensure their safe and effective use. However, chemical basis of toxicity reducing for most herbal medicines remains unclear.* Stellera chamaejasme* L., particularly the root (ruixianglangdu), is toxic. Thus, ruixianglangdu is commonly processed with vinegar or milk to reduce toxicity. Here, with help of multiple-ion monitoring (MIM)-based metabolomics, we comprehensively capture chemical information of ruixianglangdu. Then, 33 differential components between crude drugs and processed products were identified or tentatively characterized by multiple-ion monitoring–information dependent acquiring–enhanced product ion (MIM-IDA-EPI), whose level changed after being processed by vinegar or milk. It was found that flavonoids especially biflavonones could be the important chemical basis of toxicity reduction for processed ruixianglangdu. In addition, some coumarins and lignanoids could also play a role in reducing toxicity. It is believed that MIM-based metabolomics method was valuable for exploring chemical basis of toxicity reduction for processing. The data is critical to further study the mechanism of toxicity reducing for processed ruixianglangdu.

## 1. Introduction

Processing of herbal medicinal materials is a pharmaceutical technique based on traditional Chinese medicine (TCM) theory, which aimed to enhance the efficacy and/or reduce the toxicity of crude drugs [1]. For herbal medicines, many processing approaches could be used to reduce the toxicity of crude drugs based on different clinical experience. However, chemical basis of toxicity reduction for most herbal medicines remains unclear. Because of multiple components in herbal medicines, exploring chemical basis is extensively dampened by comprehensively and accurately capture information in complex multicomponent samples.

*Stellera chamaejasme* L. is a perennial herb belonging to the Thymelaeaceae family of flowering plants, native to Russia, China, and Mongolia [2]. Ruixianglangdu, roots of* Stellera chamaejasme* L. (ruixianglangdu), is one of the TCM most commonly used as a remedy for furuncle carbuncle and tuberculosis of the lymph nodes, with varied biological activity, including antiviral, antitumor, antibacterial, and anti-inflammatory activities [3–5]. Ruixianglangdu could poison or even kill the cattle if ingested by mistake [6]. By now, some processing methods have been used to attenuate toxicity of ruixianglangdu. Processing with milk is the special processing method of Mongolian medicine [7]. Ruixianglangdu treated with an optimum amount of vinegar is the processing method described in Pharmacopoeia [8]. They are all the representative processing methods of ruixianglangdu.

Current methods including thin layer chromatography (TLC) and high-performance liquid chromatography-ultraviolet spectrophotometry (HPLC-UV) used for the determination of multiple components in ruixianglangdu and its processed products are not enough selective and accurate [9, 10]. They only concentrated on total component or several components, which could not comprehensively explore the changes of multiple components. For HPLC, the analytical time was long and the peak resolution was not well to determine multiple components. Meanwhile, the HPLC-UV analysis of multiple components is affected by low sensitivity and low signal-to-noise ratio. Hence, the ultraperformance liquid chromatography–triple quadrupole tandem mass spectrometry (UPLC-MS/MS) method, using different multiple reaction monitor (MRM) channels at the same time, would be theoretically suitable for simultaneous quantification of multiple components in complex matrix. Multiple-ion monitoring (MIM) can be used in the cases in which the MS/MS diagnostic fragment is the same, in terms of intensity and selectivity of the parent ion [11, 12]. By now, the metabolomics approach using MIM has been successfully applied to discover biomarkers in plasma, rice, and so on [13, 14]. In addition, the MIM has also been used to determine some compounds in biological samples, such as bicyclol and peptides, with acceptable accuracy, sensitivity, and selectivity [15, 16].

In this study, a MIM-based metabolomics method was applied to explore chemical basis of toxicity reducing for processed ruixianglangdu using LC-MS/MS equipped with an ESI source. Here, we used raw ruixianglangdu and two different procedures for processing ruixianglangdu (processed with vinegar or milk), as examples. Three steps are included to explore the chemical basis of toxicity reduction: (1) MIM-based metabolomics was applied for comprehensively capturing chemical information of samples; (2) the analysis of the differential component between the crude drugs and the products was performed by multiple-ion monitoring–information dependent acquiring–enhanced product ion (MIM-IDA-EPI).

## 2. Experimental

### 2.1. Reagents and Chemicals

Neochamaejasmin A was purchased from Shanghai Jianglai Biological Technology Co., Ltd. (Shanghai, China). Neochamaejasmin B, isochamaejasmin, and daphnoretin were purchased from Chengdu Chroma-Biotechnology Co., Ltd. (Sichuan, China). The purity of the four standards was > 95%. Glutamic acid, tyrosine, isoleucine, leucine, phenylalanine, and tryptophan were purchased from the National Institutes for Food and Drug Control (Beijing, China). HPLC-grade acetonitrile from Honeywell (Morris, America) and formic acid (HPLC grade) from Sigma-Aldrich (Steinheim, Germany) were purchased. Rice vinegar and fresh milk were obtained from Foshan Haitian Flavoring & Food Co. Ltd. (China) and Yili (Inner Mongolia Yili Industrial Group Co., Ltd., China), respectively. Ultrapure water was prepared using a Milli-Q SP system (Millipore, Bedford, MA, USA).

### 2.2. Plant Materials and Sample Preparation

Ruixianglangdu was purchased in Anguo (Hebei, China) and identified using morphological and histological methods by Dr. Guihua Cui, which was deposited at Institute of Chinese Materia Medica. Raw ruixianglangdu was processed by vinegar or milk to obtain 3 processed products. 350 g of raw ruixianglangdu was mixed with rice vinegar or fresh milk, left to soak for 6 h, then boiled for 30 min, and eventually dried. Ultimately, 6 batches of samples were obtained for subsequent analysis.

According to the usage of TCM, the raw or processed products (50 g) were boiled for 30 min with 500 mL water, and most solvent was removed through boiling. Then the whole supernatant was collected, diluted to 100 mL with water, and passed through a 0.22 mm filter. The filtrate was stored at 4°C in a refrigerator before the LC-MS analysis.

### 2.3. LC-MS Analysis

A Waters ACQUITY UPLC–QTRAP™ 5500 mass spectrometer was used to perform multiple-ion monitoring–information dependent acquiring–enhanced product ion (MIM-IDA-EPI) and MIM. A Waters ACQUITY UPLC (Waters, USA) equipped with a binary solvent delivery system, an autosampler, and a column compartment was used. Detection was performed using a QTRAP™ 5500 system from Applied Biosystems/MDS Sciex (Applied Biosystems, Foster City, CA, USA), a hybrid triple quadrupole linear ion trap mass spectrometer. The instrument was operated using an electrospray ionization source (ESI). The ion spray voltage was set to 5.5 kV, and the turbo spray temperature was maintained at 550°C. Nebulizer gas (gas 1) and heater gas (gas 2) were set at 50 and 50 psi, respectively. The curtain gas was kept at 35 psi and the interface heater was on. Nitrogen was used as nebulizer and auxiliary gas. Stepwise MIM in positive and negative scan was adopted, with 3 scans ranging from* m/z *51 to* m/z *350, from* m/z *351 to* m/z *650, and from* m/z *651 to* m/z *950, respectively, with mass step 1.0 Da and dwell time 5 msec. Three hundred MIM transitions in a single run were obtained. The declustering potential (DP) and collision energy (CE) of each MIM were set at 60 V and 5 eV, respectively. For obtaining fragmentation ions, all the ions exceeding 5000 cps were used to trigger the acquisition of EPI spectra. The MS scan function was controlled with the Analyst software (versions 1.6.2) from Applied Biosystems/MDS Sciex.

The chromatographic separation was performed on a Waters HSS C_18_ column (2.1 × 100 mm, 1.8 *μ*m) with the column temperature set at 40°C. The mobile phase consisted of water containing 0.1% formic acid (A) and only acetonitrile (B), with a linear gradient elution at a flow rate of 0.3mL/min. The gradient program was as follows: 99-70% A (0-10 min); 70-55% A (10-25 min); 1% A (25-30 min); 99% A (30-33 min). The volume of the sample injection was 1 *μ*L.

### 2.4. Statistical Data Analysis

The scores plot of PCA was drawn using SIMCA-P version 12.0 software (Umetrics AB, Umea, Sweden). Furthermore, an independent T-test (*p*<0.01) (Microsoft Office Excel 2010) was used to determine if the change in processing was statistically different at the univariate analysis level. Furthermore, an independent T-test (*p*<0.01) (Microsoft Office Excel 2010) was used to analyze the differential component between raw and milk-processed ruixianglangdu. Meanwhile, the differential component between raw and vinegar-processed ruixianglangdu was also analyzed with T-test. Components with statistical difference were marked with asterisk.

## 3. Results and Discussion

### 3.1. Metabolic Profiling of Ruixianglangdu

**Figure 1:**
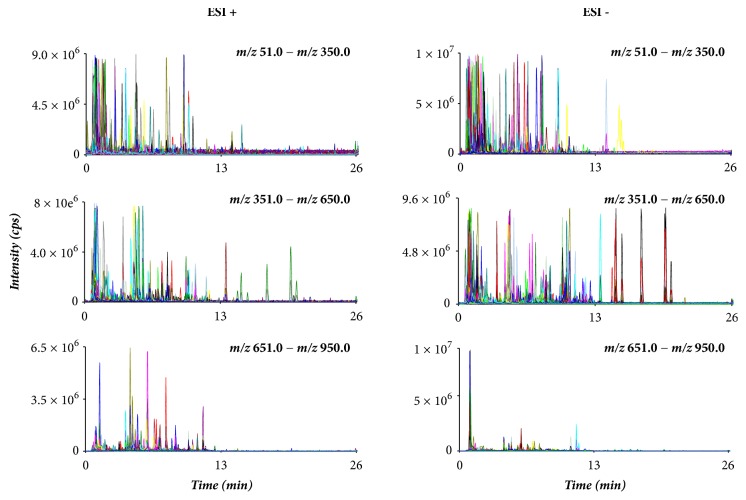
Stepwise multiple-ion monitoring (MIM) chromatogram of ruixianglangdu.

**Figure 2:**
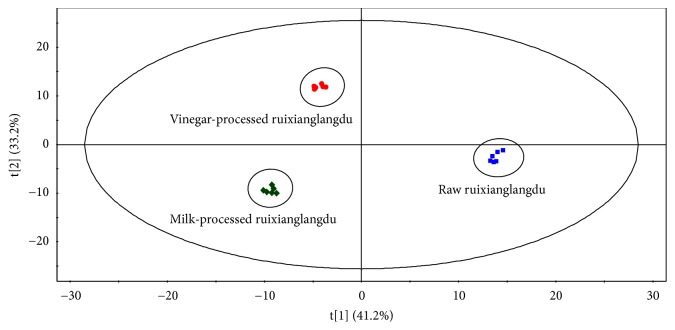
Principle component analysis scores plot for ruixianglangdu and its processed products.

Global profiling of stepwise scan MIM in positive and negative mode was adopted to analyze 6 batches of raw, vinegar-processed, and milk-processed ruixianglangdu, respectively. The chromatogram of ruixianglangdu is shown in Figure 1. On the chromatogram, the ion peaks were easy to detect and their peak areas could be calculated by Analyst software. The ion peaks in the scan range from* m/z *651 to* m/z *950 was relatively less than those in the scan range from* m/z *51 to* m/z *650, and most components were eluted before 13 min. It was suggested that most of the compounds are hydrophilic with higher polarity. A total of 1175 ions with signal-to-noise ratio (S/N) > 10 were obtained, including 563 ions in positive mode and 612 ions in negative mode. The resultant 2D matrices, including paired* m/z*–retention time, sample names, and peak areas of 1175 ions, were analyzed by PCA to visualize general clustering and trends. As shown in Figure 2, an obvious separation trend can be observed between ruixianglangdu and its processed products. And the 2 kinds of processed products were also obviously separated in the PCA.

### 3.2. Identification of Components That Changed in Processing

After filtering the fragment ions and isotope ions, 37 components found in positive and 26 components found in negative mode were screened out, which differ after processing with milk or vinegar. The fragmentation profile of changed constituents was rapidly analyzed using MIM-IDA-EPI. Eventually, 33 compounds were identified or tentatively characterized in ruixianglangdu, whose level changed after being processed by vinegar or milk. These components involved 14 flavonoids, 5 coumarins, 8 lignanoids, 5 amino acids, and 1 diterpenoid. Their detailed information is presented in Table 1, such as adduct ion, fragmentation ions of MS^n^, and retention time. By now, 22 compounds had been isolated and identified from ruixianglangdu [2, 17]. Compounds 9, 10, 12, 19, and 28-32 could be unambiguously identified as neochamaejasmin A, neochamaejasmin B, isochamaejasmin, daphnoretin, glutamic acid, tyrosine, isoleucine, leucine, and phenylalanine, respectively, by comparing their retention times, adduct ions, and product ions with authentic standards. A detailed description of structural characterization process was as follows: 

*Flavonoids *compound 1 was identified as epiafzelechin-7-O-glucoside with [M+H]^+^* m/z* 437.1, a series of fragment ions [M+H-Glc]^+^* m/z* 275.2, [M+H-H_2_O-Glc-H_2_O]^+^* m/z* 257.2, and characteristic fragments of RDA cleavage m/z 139.0. In the MS^2^ spectrum of flavonoids (nos. 2 and 4), the ion at m/z 705.18 fragmented to ion [M+H-Glc]^+^* m/z *543.1. Considering that isochamaejasmin-7-O-glucoside has been isolated from roots of* Stellera chamaejasme*, compounds 2 and 4 were tentatively identified as isochamaejasmin/chamaejasmine/neochamaejasmin A/neochamaejasmin B-7-O-glucoside. Based on [M+H]^+^* m/z *275.1, a series of fragment ions [M+H-H_2_O]^+^* m/z* 257.2, and characteristic fragments of RDA cleavage* m/z *139.0, compound 3 was identified as epiafzelechin. Compounds 5 and 6 were tentatively identified as naringenin-epiafzelechin or naringenin-afzelechin in agreement with [M+H]^+^* m/z* 545.1. Their MS^2^ spectrum gave the fragment ions m/z 409.1, with fragments of RDA cleavage* m/z* 153.0. Compound 7 exhibited the deprotonated molecule [M+H]^+^* m/z* 341.1. Major fragment ions [M+H-H_2_O]^+^* m/z* 323.2, [M+H-C_5_H_9_]^+^* m/z* 271.2, a fragment of RDA cleavage* m/z* 137.0 were shown in the MS/MS spectrum. Thus, it was tentatively identified as 8-prenylnaringenin. In the MS^2^ spectrum of compound 8, the ion at* m/z *543.1 fragmented to the ion* m/z *345.1, losing one molecule of bis(4-hydroxyphenyl)methane, and* m/z* 199.2. It was identified as chamaechromone. Four biflavonones (nos. 9, 10, 11, and 12) had a series of the same fragment ions* m/z* 449.0, 391.1, 311.2, and 153.0. The peak at* m/z *391.1 and 153.0 corresponded to the RDA cleavage. Compounds nos. 9, 10, and 12 were confirmed as neochamaejasmin A, neochamaejasmin B, and isochamaejasmin by comparison with standards. Compound 11 was identified as chamaejasmine due to the similar fragment ions with compounds 9, 10, and 12. Compound 13 exhibited [M+H]^+^* m/z* 557.1, major fragment ions* m/z *449.0, and characteristic fragments* m/z *153.0, which was similar to compounds 9-12, which was identified as 7-methoxylneochaejasmin A. Compound 14 had a series fragment ions* m/z* 445.1, 419.2, 355.1, and 337.2. The peak at* m/z *419.2 corresponded to the RDA cleavage. Compound 14 was tentatively identified as chamaejasmenin A. All the 14 flavonoids except for compound 7 had been isolated from roots of* Stellera chamaejasme* L. [17–23]. 

*Coumarins *compound 15 exhibited [M+H]^+^* m/z* 193.1. Based on major fragment ions [M+H-CH_3_OH]^+^* m/z* 161.0, [M+H-CH_3_OH-CO]^+^* m/z* 133.0, and [M+H-CH_3_OH-2CO]^+^* m/z* 105.0, this compound was tentatively assigned to scopoletin. Compound 16 was tentatively identified as umbelliferone glucoside, with a series of fragment ions [M+H-Glc]^+^* m/z* 163.0 and [M+H-Glc-CO_2_]^+^* m/z* 119.1. Compound 17 was identified as daphnetin with [M+H]^+^* m/z* 177.0 and a series of fragment ions [M-H-CO]^−^* m/z* 149.0, [M-H-CO_2_]^−^* m/z* 133.0, and [M-H-CO-CO_2_]^−^* m/z* 105.1. The MS^2^ spectrum of compound 18 with [M-H]^−^* m/z* 161.0 gave the characteristic fragment ions of* m/z* 133.1, 117.0, and 105.1, which corresponded to [M-H-CO]^−^, [M-H-CO_2_]^−^, and [M-H-2CO]^−^. It was tentatively identified as umbelliferone. The MS/MS mass spectrum of compound 19 [M+H]^+^* m/z *353.1 presented the specific fragments of [M+H-CH_3_]^+^* m/z* 338.2. It was confirmed as daphnoretin by comparison with standard. All the 5 coumarins had been isolated from roots of* Stellera chamaejasme* L. [21, 24, 25].

*Lignanoids *base peak of compounds 20 and 22 was* m/z* 359.2. Compounds 20 and 22 were tentatively identified as isolariciresinol-4'-O-glucoside and isolariciresinol-9-O-glucoside, based on their polarity. Two compounds (no. 21 and 23) had the same [M-H]^−^* m/z* 523.2 and characteristic fragment [M-H-Glc]^+^* m/z* 361.3. Based on their different polarity, compounds 21 and 23 were tentatively identified as secoisolariciresinol-4-O-glucoside and secoisolariciresinol-9-O-glucoside, respectively, based on their polarity. Based on the characteristic fragment [M-H-Glc]^−^* m/z *357.2, compound 24 with [M-H]^−^* m/z* 519.2 was tentatively identified as pinoresinol-4-O-glucoside. Compound 25 was tentatively identified as aschantin with a series of fragment ions [M+H-H_2_O]^+^* m/z* 383.2 and characteristic fragment ion* m/z* 167.2. Compound 26 with [M+H]^+^* m/z* 419.2 was tentatively identified as syringaresinol, which fragmented to ion [M+H-H_2_O]^+^* m/z* 401.1 and [M+H-HOCH_3_]^+^* m/z* 387.1. Compound 27 was tentatively identified as pinoresinol with [M+H]^+^* m/z* 359.1 and a series of fragment ions [M+H-H_2_O]^+^* m/z* 341.2, [M+H-2H_2_O]^+^* m/z* 323.2, and characteristic fragment ion m/z 137.0. Only 3* lignanoids* (no. 24, 26, and 27) had been isolated from roots of* Stellera chamaejasme* L. [21, 26].

The 5 amino acids compounds (28-32) were confirmed by comparison with standard. Compound 33 was tentatively identified as stelleramacrin B with [M+H]^+^* m/z* 613.3 and a characteristic fragment ion [M+H-C_14_H_24_-H_2_O]^+^* m/z* 405.2, which had been isolated from roots of* Stellera chamaejasme* L. [20].

### 3.3. Exploring Chemical Basis of Toxicity reducing for Processing

**Figure 3:**
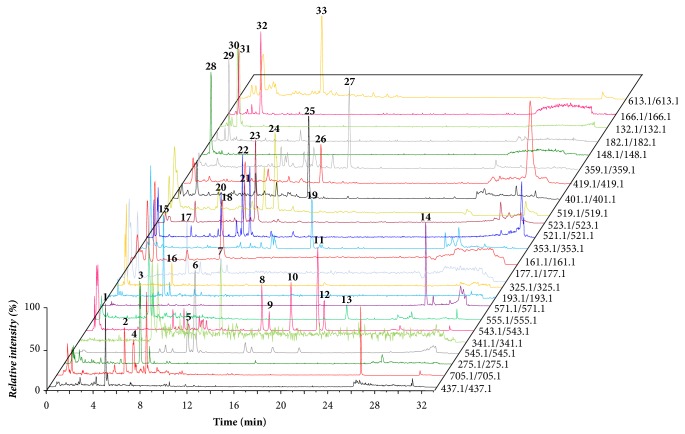
The extracted ion chromatogram (XIC) of the 33 components found to be significantly altered after being processed by vinegar or milk. All the ion pairs are monitored in positive mode except for 177.1/177.1, 161.1/161.1, 521.1/521.1, 523.1/523.1, 519.1/519.1, and 613.1/613.1. Compound number is consistent with the compound number in Table 1.

**Figure 4:**
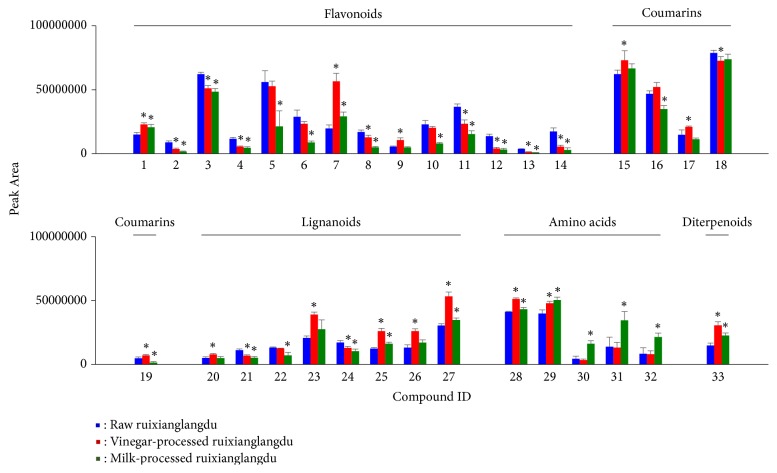
Peak area of 33 compounds changed in processed ruixianglangdu. Compound ID is consistent with the number in Table 1. Compounds with statistical difference between raw and milk/vinegar-processed ruixianglangdu were marked with asterisk (*∗*).

The extracted ion chromatogram (XIC) of the 33 components was found to be significantly altered after being processed by vinegar or milk as shown in Figure 3. T-test of the 33 components between raw and milk/vinegar-processed ruixianglangdu was performed. As shown in Figure 4, when the raw ruixianglangdu was processed by vinegar, 25 components exhibited significant changes, including 11 flavonoids (no. 1-4, 7-9, and 11-14), 4 coumarins (no. 15 and 17-19), 7 lignanoids (no. 20-21 and 23-27), 2 amino acids (no. 28 and 29), and 1 diterpenoid. Eleven of the 25 components underwent a concentration decrease after vinegar processing, including 8 flavonoids (no. 2-4, 8, 11-14), 1 coumarin (no. 18), and 2 lignanoids (no. 21 and 24). The change ratio of components in raw to vinegar-processed ruixianglangdu was in the range 0.3~2.9. When raw ruixianglangdu was processed by milk, 26 components exhibited significant changes, including 13 flavonoids (no. 1-8, 10-14), 2 coumarins (no. 16 and 19), 5 lignanoids (no. 21, 22, 24, 25, and 27), 5 amino acids (no. 28-30), and 1 diterpenoid. Sixteen of the 26 components exhibited decreasing tendency, including 11 flavonoids (no. 2-6, 7, 10-14), 2 coumarins (no. 16 and 19), and 3 lignanoids (no. 21, 22, and 24). It is worth noting that 10 components underwent a concentration decrease after processing with both milk and vinegar, including 8 flavonoids (chamaejasmine-7-O-glucoside, epiafzelechin, neochamaejasmin A-7-O-glucopyranoside, chamaechromone, chamaejasmine, isochamaejasmin, 7-methoxylneochaejasmin A, and chamaejasmenin A) and 2 lignanoids. Decreased flavonoid could be due to the thermic treatment performed during the raw ruixianglangdu extraction. For example, total flavonoid content of sea buckthorn extract reduction of 90.04% was observed as a result of heating at 100°C after 25 min [27]. The total and five flavonoids were also significantly decreased after fresh Cara Cara juice heating at 90°C for 120 min [28]. Quercetin was also unstable in aqueous solution, and aqueous stability of flavonol was associated with B-ring substitution [29].

Total flavonoids obtained from* Stellera chamaejasme* have showed strong toxicity to mice and rabbit [30]. Chamaechromone (no. 8) and isochamaejasmin (no. 12) were major components in ruixianglangdu with cytotoxicity to canine kidney MDCK cells [31, 32]. Coumarin daphnoretin (no. 19) was found to be a cytotoxic constituent of* Dirca occidentalis* (Thymelaeaceae) [33]. Based on these changing components and literature, for the processed ruixianglangdu, flavonoids especially biflavonones could be the important chemical basis of toxicity reduction. In addition, some coumarins and lignanoids that decreased after processing could also play a role in reducing toxicity.

Compared with milk-processed ruixianglangdu groups, the 10 components involving 8 flavonoids had a lower level in the vinegar-processed ruixianglangdu group. It was inferred that processing method with milk could be better for toxicity reduction of ruixianglangdu. According to literature, the mortality of zebrafish embryo was higher after treating with vinegar-processed ruixianglangdu than milk-processed ruixianglangdu [34], which was consistent with our inference. 

## 4. Conclusions

With the help of MIM-based metabolomics, this study illustrated the chemical basis of toxicity reducing for processed ruixianglangdu. Flavonoids especially biflavonones could be the important chemical basis of toxicity reducing. In addition, some coumarins and lignanoids that decreased after processing could also play a role in reducing toxicity. Compared with vinegar processing, processing method with milk could be better for toxicity reduction of ruixianglangdu. It is believed that MIM-based metabolomics method was valuable for exploring chemical basis of toxicity reduction for processed TCM. The data is critical to further study the toxicity reduction mechanism of processed ruixianglangdu.

## Figures and Tables

**Table 1:** Identification of compounds in ruixianglangdu.

No.^a^	t_R_ (min)	Parent ion *[M+H]*^+^ or *[M-H]*^−^ (*m/z*)	Molecular formula	Fragmentation profile (*m/z*) (Relative abundance %)	Identification
*Flavonoids (parent ions: [M+H]* ^+^)					

1*∗*	4.81	437.1	C_21_H_24_O_10_	275.2, 257.2, 149.0, 139.0 (100%), 107.0	Epiafzelechin-7-O- gluconoside
2*∗*	5.85	705.2	C_36_H_32_O_15_	543.1 (100%)	Isochamaejasmine/ Chamaejasmine/ Neochamaejasmine A/ Neochamaejasmine B-7-O- gluconoside
3*∗*	6.41	275.1	C_15_H_14_O_5_	257.2 (100%), 139.0	Epiafzelechin
4*∗*	6.63	705.2	C_36_H_32_O_15_	543.2 (100%), 527.1, 311.2	Same as no. 2
5*∗*	9.84	545.1	C_30_H_24_O_10_	527.2, 409.1 (100%), 283.2, 153.0	Naringenin-epiafzelechin or Naringenin-afzelechin
6*∗*	10.48	545.1	C_30_H_24_O_10_	527.2, 409.1 (100%), 283.2, 153.0	Same as no. 5
7	12.13	341.1	C_20_H_20_O_5_	323.2 (100%), 291.1, 271.2, 187.2, 137.0	8-Prenylnaringenin
8*∗*	14.83	543.1	C_30_H_22_O_10_	345.1, 199.2 (100%)	Chamaechromone
9*∗*	15.38	543.1	C_30_H_22_O_10_	449.0, 391.1, 311.2, 199.1, 153.0 (100%)	Neochamaejasmine A
10*∗*	17.19	543.1	C_30_H_22_O_10_	449.0, 417.2, 391.1, 311.2, 231.1, 199.2, 153.0 (100%)	Neochamaejasmine B
11*∗*	19.47	543.1	C_30_H_22_O_10_	449.0, 417.2, 391.1, 311.2, 153.0 (100%)	Chamaejasmine
12*∗*	20.04	543.1	C_30_H_22_O_10_	449.0, 417.2, 391.1, 311.2, 153.0 (100%)	Isochamaejasmine
13*∗*	21.35	557.1	C_31_H_24_O_10_	449.0, 153.0 (100%)	7-Methoxylneochaejasmin A
14*∗*	27.63	571.2	C_32_H_26_O_10_	463.1, 445.1, 419.2 (100%), 355.1, 337.2, 245.2, 227.3	Chamaejasmenin A

*Coumarins (parent ions of compounds 15, 16 and 19: [M+H]* ^+^ *, parent ions of compounds 17 and 18: [M-H]* ^−^)					

15*∗*	4.81	193.1	C_10_H_8_O_4_	161.0 (100%), 133.0, 115.0, 105.0, 76.5	Scopoletin
16*∗*	4.89	325.1	C_15_H_16_O_8_	280.2, 163.0 (100%), 119.0, 107.0	Umbelliferone glucoside
17*∗*	5.85	177.0	C_9_H_6_O_4_	149.0 (100%), 133.0, 121.1, 105.1, 93.0, 77.0	Daphnetin
18*∗*	7.88	161.0	C_9_H_6_O_3_	133.1 (100%), 117.0, 105.1, 89.0, 77.0	Umbelliferone
19*∗*	14.44	353.1	C_19_H_12_O_7_	338.2, 267.1, 251.2, 179.2 (100%)	Daphnoretin

*Lignanoids (parent ions of compounds 25-27: [M+H]* ^+^ *, parent ions of compounds 20-24: [M-H]* ^−^)					

20	6.21	521.2	C_26_H_34_O_11_	359.2 (100%), 159.1	Isolariciresinol-4'-O- glucoside
21	7.46	523.2	C_26_H_36_O_11_	361.3 (100%), 165.2, 136.2	Secoisolariciresinol-4-O- glucoside
22	7.98	521.2	C_26_H_34_O_11_	359.2, 329.3 (100%),178.1	Isolariciresinol-9-O- glucoside
23	8.41	523.2	C_26_H_36_O_11_	361.3 (100%), 313.2	Secoisolariciresinol-9-O- glucoside
24*∗*	9.69	519.2	C_26_H_32_O_11_	357.2 (100%)	Pinoresinol-4-O-glucoside
25	11.64	401.2	C_22_H_24_O_7_	383.2, 217.2, 207.2, 167.2, 135.0(100%)	Aschantin
26*∗*	11.76	419.2	C_22_H_26_O_8_	401.1, 387.1, 236.1 (100%), 182.1	Syringaresinol
27*∗*	13.42	359.1	C_20_H_22_O_6_	341.2, 323.2, 311.2, 137.0 (100%)	Pinoresinol

*Amino acids (parent ions: [M+H]* ^+^)					

28	0.92	148.1	C_5_H_9_NO_4_	130.0 (100%), 102.0	Glutamic acid
29	1.99	182.1	C_9_H_11_NO_3_	165.0 (100%), 147.0, 136.0, 123.0	Tyrosine
30	2.05	132.1	C_6_H_13_NO_2_	86.0 (100%)	Isoleucine
31	2.21	132.1	C_6_H_13_NO_2_	114.0, 86.0 (100%)	Leucine
32	3.05	166.1	C_9_H_11_NO_2_	149.0, 120.0 (100%), 103.0	Phenylalanine

*Diterpenoids (parent ions: [M-H]* ^−^)					

33*∗*	7.18	613.3	C_35_H_50_O_9_	567.2, 405.2, 309.2, 179.0 (100%), 119.2, 113.0	Stelleramacrin B

a: The compounds isolated from ruixianglangdu are marked with *∗*.

## Data Availability

The data supporting the findings of this study are available within the article. Raw data and additional information of this study are available from the corresponding author on request.
